# I am the best and I have everything in life

**DOI:** 10.1192/j.eurpsy.2022.1559

**Published:** 2022-09-01

**Authors:** C. Vallecillo Adame, T. Jiménez Aparicio, C. De Andrés Lobo, A. Gonzaga Ramírez, M. Queipo De Llano De La Viuda, G. Guerra Valera, I. Santos Carrasco, J. Gonçalves Cerejeira, M. Fernández Lozano, M.J. Mateos Sexmero, B. Rodríguez Rodríguez, N. Navarro Barriga, N. De Uribe Viloria

**Affiliations:** 1 Hospital Clínico Universitario, Psiquiatría, Valladolid, Spain; 2 Hospital Clínico Universitario de Valladolid, Psiquiatría, VALLADOLID, Spain; 3 Hospital Clínico Universitario de Valladolid, Psychiatry, Valladolid, Spain; 4 Clinical Hospital of Valladolid, Psychiatry, Valladolid, Spain; 5 Hospital Universitario Fundación de Alcorcón, Psychiatry, Madrid, Spain

**Keywords:** borderline intelligence, megalomaniacal ideas, psychology, Psychosis

## Abstract

**Introduction:**

Megalomanic ideas in a patient with limited intellectual functioning may be due to the psychotic clinic or be the result of their disability.

**Objectives:**

This case is intended to highlight the importance of a joint approach between psychiatrists and psychologists to assess functionality before and after the psychotic episode.

**Methods:**

34-year-old woman with no mental health history. She came to the emergency department for an episode of aggression at home. Her parents report that they have observed strange behaviour, she is more aggressive, speaks alone, changing voices and global insomnia in the last few days. Her language is incoherent and disorganised, with a long response latency. Megalomaniacal and catastrophic delusions. Possible auditory hallucinations and thought control phenomena.

**Results:**

During admission, antipsychotic treatment was introduced with good tolerance and response on the part of the patient. She has been distancing herself from the ideas and has become somewhat critical. A psychological evaluation was carried out with different scales that showed borderline IQ.

**Conclusions:**

It is important to make a good assessment of the patient’s symptoms in order to make a differential diagnosis. In this case, it is advisable to carry out a control and follow-up, as well as a neuropsychological assessment before and after the acute episode. In addition, a multidisciplinary approach with psychologists, psychiatrists and social workers is important.

**Disclosure:**

No significant relationships.

